# The Spirometer

**Published:** 1851-02

**Authors:** 


*The Spirometer.* Clinical Lecture. By Prof. S. Jackson.

Medical Science finds, in almost every department of knowledge, some portion of its facts or laws applicable to itself, and lays them under contribution for its own advancement, or the augmentation of its resources.

The introduction of physics into the practice of medicine, applied to the diseases of the thoracic organs, belongs to the present time, and is the most valuable improvement that has yet been made in the diagnosis of disease.

Percussion and auscultation are means of ascertaining and interpreting the physical causes of sounds which can be determined by them as belonging to the thorax and its contents.

Skill in these processes imparts a degree of certainty to the diagnosis of thoracic affections, that nearly reaches perfection; it almost equals that of ocular inspection. There is, however, this defect attending them; disease must have made some progress, and change of structure have taken place to a certain extent, before physical pathological signs, that is, alteration in the normal sounds, or production of abnormal sounds would be produced. They do not avail us in indicating the approach of disease, or its forming stages, except to a very limited extent.

Another contribution from the domain of physics has been made, by Mr. Hutchinson, to the investigation of the respiratory functions in health and disease. It consists in an instrument he has invented, by which may be measured the amount of air that can be taken into and expelled from the lungs by voluntary effort; or what he calls "the vital capacity" of the lungs. By this instrument Mr. Hutchinson believes that incipient disease may be detected before physical signs exist. This instrument he names spirometer.

On the table is an instrument of the kind. It is simple and less expensive than that of Mr. Hutchinson. It was planned by a gentleman of this city, Mr. Charles McEuen, who has been confined to his room for some months by a pulmonary affection; possessing an active mind, with a turn for philosophical pursuits, he occupies his time in scientific observations and investigations. I gave him Mr. Hutchinson's paper, published in the Medico Chirurgical Transactions, containing a diagram of his instrument. Mr. McEuen constructed the instrument now before you on the same principles. I think it preferable to the original.

The instrument will be seen to consist of a cylinder containing water, in which is immersed another cylinder inverted, into whicn the expired air finds its way. This cylinder is counterpoised by a weight attached to a cord passing over a wheel of large diameter, and which rotates with the ascent of the cylinder, caused by the entrance of the expired air, and on which a scale indicates the amount that has been introduced.

The person using this instrument must loosen any part of his dress that may restrain the movements of the chest or abdomen. He then deliberately expands his chest to its greatest extent, and expires through the mouth piece and air-tube into the cylinder. As this rises the wheel turns round, and an index marks on the scale, in inches, the amount expired.

To understand the use of this instrument, it is requisite you should possess some preliminary information on the respiratory actions, and to what extent they influence the air in the lungs.

Inspiration and expiration are performed by muscular power, and are both voluntary and involuntary actions. The extent to which they may be carried varies in different individuals, and in the same individual at different times. They have a limit which cannot be surpassed; the lungs can never be emptied, by the most strenuous efforts of expiration.

The air in the lungs is, therefore, divisible into two portions. The first, which is a fixed quantity, is that over which the will has no control, but remains after the strongest expiration, and is contained in healthy lungs after death. Its amount must correspond with the size of the thorax. Mr. Hutchinson calls this the residual air.

The second portion is that which is controlled by the will and muscular action. This portion Mr. Hutchinson divides into three sub-portions. 1st. Reserve air, or that portion which, after an ordinary expiration, may still be thrown out by a voluntary effort. 2d. Breathing air, or the portion inhaled and exhaled in ordinary breathing, when at rest; and 3d. Complemental air, or that portion that can be inhaled, by the strongest effort, beyond the amount of ordinary inspiration.

The three last are included in, and designated by the term "Vital Capacity." It is, in fact, the highest effort of the muscles producing respiration. The spirometer measures the "vital capacity" of an individual, and, it appears to me, is the measure ef the muscular respiratory power.

Mr. Hutchinson was struck with the fact, that the vital capacity had no relation to the size of the thorax. On the contrary, he found, by experiment, that persons of the largest thorax possessed a less vital capacity than others with chests much smaller.

In the course of his observations he remarked that there appeared to prevail a very close relation between the height of individuals and their vital capacity. This circumstance was the more strange and unaccountable, as height depends most commonly on the length of the lower extremities, and not on that of the chest or trunk alone.

From observation made on a large number of individuals, taken indiscriminately from various classes of society, amounting to 2150, he arrived at the conclusion, that the vital capacity is a constant quantity, and holds a close relation with the height.

From the result of direct examination, in near 2,000 cases, Mr. Hutchinson felt authorized to announce the following rule, "For every inch of height (from 5 feet to 6 feet) eight additional cubic inches of air, at 60 deg. are given out by a forced expiration."

He further states, "here is a guide for the operator, and a rule given that will enable us to compare men of different stature and conditions of health, one with another."

If this result should be found accurate, the spirometer would be unquestionably a most valuable addition, to aid the physician in deciding the state of health, in many cases, that are, by our common mode of examination, enveloped in great uncertainty.

The following table shows the relation between height and vital capacity.

| Height. | | | | | | Total Capacity. |
|---|---|---|---|---|---|---|
| Ft. | In. | | Ft. | In. | | Cubic Inches. |
| 5 | 0 | to | 5 | 1 | - - - | 174 |
| 5 | 1 | " | 5 | 2 | - - - | 182 |
| 5 | 2 | " | 5 | 3 | - - - | 190 |
| 5 | 3 | " | 5 | 4 | - - - | 198 |
| 5 | 4 | " | 5 | 5 | - - - | 206 |
| 5 | 5 | " | 5 | 6 | - - - | 214 |
| 5 | 6 | " | 5 | 7 | - - - | 222 |
| 5 | 7 | " | 5 | 8 | - - - | 230 |
| 5 | 8 | " | 5 | 9 | - - - | 238 |
| 5 | 9 | " | 5 | 10 | - - - | 246 |
| 5 | 10 | " | 5 | 11 | - - - | 254 |
| 5 | 11 | " | 6 | 0 | - - - | 262 |

Before making any further comment on the rule laid down authoritatively by Mr. Hutchinson, I will test by the instrument the vital capacity of some patients affected with pulmonary disease, who are now present.

(Several patients, cases of chronic pleurisy, phthisis pulmonalis in various stages, and emphysema, were tested, the height and age being first ascertained.)

They vary, you perceive, from 80 to 120 cubic inches expired. Not one of the above patients approaches to the normal vital capacity, in accordance with his height and age.

They are from 80 to 200 cubic inches below tho standard according to the table.

I must confess, that I have some misgivings as to the accuracy of this rule, and cannot but suspect that another element than that of height regulates the extent of vital capacity, and that element is the muscular force of the respiratory muscles.

I express this only as a suspicion. The extent of Mr. Hutchinson's inquiries, the evident care, labor and conscientiousness with which he pursued his investigations, entitle them to the highest consideration, and they should not be lightly questioned.

But, in a considerable number of examinations I have made on healthy individuals, of the same height and age, with slight difference of weight, there is manifest such wide difference of vital capacity, that I cannot but hesitate in adopting the rule as universally applicable.

I have, for instance, examined, within 24 hours, three gentlemen in perfect health, one a member of our profession, who have all been and are engaged in active pursuits. They are, respectively, 5 feet 11 inches, 5 feet 11½ inches, and 6 feet in height; the vital capacity of the first two is only 170 cubic inches, and of the last 190 cubic inches. According to Mr.

Hutchinson's table they ought to have a vital capacity of 250 to 260 cubic inches.

Now, these gentlemen have a peculiar, and I may say, an American conformation. I am under the impression it is not common in England. They are tall, long limbed, thin, with very slender muscles.

The highest vital capacity I have met with, is in a young gentleman 5 feet 8 inches in height, in whom it is 280 cubic inches. He is of sanguine temperament, large, bony framed, and with well developed muscles. So far as about 100 observations have been made, I have not found that uniform relation, as stated in the rule, between height and vital capacity. The differences, from 20 to 100 cubic inches, are too great to be attributed to accidental circumstances. The individuals I speak of are all in high health.

More numerous and extended observations are, however, required before a positive conclusion on this subject can be justified.

It has occurred to me that the discrepancies between Mr. Hutchinson's statements and my own observations, should they be confirmed by more numerous experiments, may depend on differences of race. The English are far more homogeneous than the Americans. In this country races are mingled, and continue to be more blended every day. As a race the English are bony, muscular and sinewy. Fxperiments with the Dynamometer have shown they possess a superiority of muscular force.

In a homogeneous population the average height and weight would be in accordance with an average development of the muscular system. But in a mixed population the same rule would not apply.

I believe there can hardly be a question as to the very marked difference in the general aspect and structure of the native born Americans, who are generally a mixed race, and those of the English, German, Irish, and French.

In examining Mr. Hutchinson's Table *A*, exhibiling the total capacity of 15 different classes, there are very striking differences to be seen. Pugilists, seamen, fire and police-men, and grenadier guards, have the greatest vital capacity. This is shown in the column of the table for the height of 5 ft. 8 in. to 5 ft. 9 in., and from 5 ft. 9 in. to 5 ft. 10 in.

*Table of the mean Vital Capacity of* 15 *different Classes.*

| | 5 ft. 8 in. to 5 ft. 9 in. | 5 ft. 9 in. to 5 ft. 10 in |
|---|---|---|
| Seamen, | 239 | 258 |
| Fire Brigade, | 231 | 237 |
| Police, Metrop., | 226 | 248 |
| Ditto Thames, | 250 | 240 |
| Paupers, | 199 | 262 |
| Mixed Class, | 238 | 246 |
| Grenadier Guards, | 233 | 240 |
| Compositors, | 214 | 231 |
| Pressmen, | 245 | 239 |
| Draymen, | 223 | 245 |
| Gentlemen, | 208 | 236 |
| Pugilists, &c., | 243 | 273 |
| Chatham Recruits, | 251 | 266 |
| Woolwich Marines, | 240 | 246 |

In this table the vital capacity certainly does not correspond to height as it respects different classes. Those classes comprehending individuals whose occupations require athletic, robust, and picked men, exhibit a vital capacity varying from 20 to 40 cubic inches higher than paupers, compositors, and gentlemen.

This table appears to sustain the conclusion which seems to follow from the observations I have made here with the Spirometer, that it is muscular power, and not height, that governs the "vital capacity."—*Med. Examiner.*

